# Tumor immune microenvironmental characteristics in Human Epidermal Growth Factor-2 (HER2) positive esophageal adenocarcinoma: A comparative analysis and biomarker study

**DOI:** 10.1016/j.tranon.2024.102079

**Published:** 2024-08-15

**Authors:** Charlotte I. Stroes, Sybren L. Meijer, Geert-Jan Creemers, Gerrit K.J. Hooijer, Nadia Haj Mohammad, Maartje Los, Marije Slingerland, Geke A.P. Hospers, Annemieke Cats, Laurens V. Beerepoot, Maarten F. Bijlsma, Hanneke W.M. van Laarhoven

**Affiliations:** a Amsterdam UMC, Location University of Amsterdam, Department of Medical Oncology, Amsterdam, the Netherlands; b Amsterdam UMC, Location University of Amsterdam, Center for Experimental and Molecular Medicine, Laboratory of Experimental Oncology and Radiobiology, Meibergdreef 9, Amsterdam, the Netherlands; c Cancer Center Amsterdam, Imaging and Biomarkers, Amsterdam, the Netherlands; d Amsterdam UMC, Location University of Amsterdam, Department of Pathology, Amsterdam, the Netherlands; e Catharina Hospital, Department of Medical Oncology, Eindhoven, the Netherlands; f Universtiy Medical Center Utrecht, Department of Medical Oncology, Utrecht University, Utrecht, the Netherlands; g Sint Antonius Hospital, Department of Medical Oncology, Nieuwegein, the Netherlands; h Leiden University Medical Center, Department of Medical Oncology, Leiden, the Netherlands; i Universtiy Medical Center Groningen, Department of Medical Oncology, University of Groningen, Groningen, the Netherlands; j Netherlands Cancer Institute, Department of Gastrointestinal Oncology, Amsterdam, the Netherlands; k Elisabeth-TweeSteden Hospital, Department of Medical Oncology, Tilburg, the Netherlands

**Keywords:** Esophageal cancer, HER2 positive tumor, Tumor immune microenvironment, Immune exhaustion, Angiogenesis

## Abstract

**Background:**

HER2 targeting in esophageal adenocarcinoma (EAC) has shown potential, but often fails to show durable response. Given the contributions of the tumor immune microenvironment (TIME) to therapeutic responses, we aimed to chart the TIME characteristics of HER2 positive tumors.

**Methods:**

84 biopsies were taken from the TRAP cohort (neoadjuvant chemoradiotherapy (nCRT) according to CROSS with trastuzumab and pertuzumab; *n* = 40; HER2^+^*n* = 40) and a control cohort with nCRT only (*n* = 44; HER2- *n* = 40, HER2^+^*n* = 4) before treatment. Biopsies were analysed using targeted gene expression analysis (Nanostring immune-oncology panel, 750 genes). Differential gene expression was assessed between HER2 positive (*n* = 44) vs. negative biopsies (*n* = 40), and non-responders (*n* = 17) vs. responders (*n* = 23) to anti-HER2 treatment. Statistical significance was determined as *p*-value <0.05, adjusted for multiple testing correction.

**Results:**

83 biopsies were eligible for analyses following quality control (TRAP cohort *n* = 40; control cohort *n* = 43); there were no significant differences in clinical characteristics between the TRAP vs. control the cohort or HER2 positive vs. HER2 negative biopsies. HER2 expression was found to associate with epithelial markers (*EPCAM p* < 0.001; *E-cadherin p* < 0.001). Moreover, HER2 expression was associated with a lower expression of immune cell infiltration, such as NK-cells (*p* < 0.001) and CD8 T-cells (*p* < 0.001), but also lower expression of immune exhaustion markers (*PDCD1LG2, CTLA4; p* < 0.001). In non-responders to anti-HER2 treatment, baseline biopsies showed increased expression of immune exhaustion markers, as well as hypoxia and VEGF signalling.

**Discussion:**

HER2 expression was associated with epithelial tumor characteristics. The HER2 positive TIME showed reduced immune cell infiltration but also lower expression of inhibitory signals associated with immune exhaustion, questioning the mechanism behind potential clinical benefit of co-administration of anti-HER2 agents and checkpoint inhibitors. As limited response was associated with increased VEGF signalling, studies could investigate potential synergism of targeting VEGF and HER2.

## Highlights

• HER2 expression was associated with epithelial markers, which have been associated with favorable outcomes.
• HER2 expression was associated with lower expression of immune cell infiltration, but also lower expression of immune exhaustion markers.
• Non-responders to HER2 targeting treatment demonstrated increased expression of immune exhaustion, as well as hypoxia and VEGF signaling.

## Introduction

Esophageal adenocarcinoma (EAC) is a major cause of mortality [1]. Currently, the incidence of esophageal cancer is over 600,000 diagnoses annually worldwide [1]. Mainly in high-income countries, a significant rise of EAC was reported throughout the last decades [1,2]. A standard treatment for locoregional disease of EAC is neoadjuvant chemoradiotherapy (nCRT) followed by resection and subsequently adjuvant nivolumab in patients with residual pathological disease [3,4]. Alternatively, perioperative chemotherapy can be considered [5,6]. Despite multimodality treatment, the prognosis of EAC remains dismal, and over 50 % of patients demonstrate recurrence of disease after treatment with curative intent [7,8].

Studies have demonstrated overexpression of HER2 (encoded by the *ERBB2* gene) in 15–22 % of EAC tumors, which can serve as a target for treatment [[9], [10], [11]]. In resectable EAC, HER2 targeted treatment has not been incorporated yet in routine practice although preliminary studies have shown benefit [12,13]. Nevertheless, a considerable number of patients fail to show durable response to HER2 targeted treatment, potentially due to intrinsic or acquired resistance mechanisms [[12], [13], [14], [15], [16]]. For instance, trastuzumab is known to exert its effects through various mechanisms, including antibody-dependent cellular cytotoxicity (ADCC). However, in gastroesophageal adenocarcinoma, the presence of HER2 expression is associated with reduced tumor immune cell infiltration. This can potentially diminish the effectiveness of trastuzumab through ADCC in these patients [[17], [18], [19]]. Improving outcomes of HER2 targeting in EAC requires further biological insight in the characteristics of the tumor immune microenvironment (TIME) of HER2 positive tumors.

We performed a translational study using 40 pre-treatment biopsies from the TRAP cohort [12]. In this study, patients with HER2 positive EAC were treated with nCRT plus trastuzumab and pertuzumab. A control cohort consisted of 44 patients treated with nCRT only. Our aims were to identify potential differences in tumor biology and associated TIME profiles between HER2 positive and HER2 negative baseline biopsies. Moreover, in an exploratory effort we aimed to identify predictive biomarkers for anti-HER2 treatment by differential gene expression analysis between responders and no-responders in the TRAP cohort.

## Materials and methods

### Patient selection and study design

This study was designed as a case-control study. Part of the samples used in this study originated from the TRAP study (NCT02120911), a phase II feasibility and biomarker study of neoadjuvant trastuzumab and pertuzumab with nCRT for the treatment of patients with resectable HER2 positive EAC [12]. HER2 positivity was defined as immunohistochemistry (IHC) 3^+^ or IHC 2^+^ with amplification of HER2, as assessed with silver in situ hybridization (ISH), while HER2 IHC 0–2^+^ without HER2 amplification was considered HER2 negative [20,21]. Baseline biopsies of all 40 patients from the TRAP study were included. In addition, archival treatment-naïve biopsies from 40 patients with HER2 negative and four patients with HER2 positive EAC receiving standard nCRT were included to allow for comparison (Supplementary Methods). Except for HER2 positivity, there were no significant differences between the control and TRAP cohort in baseline characteristics (age, sex, cTNM stage). To assess the effect of HER2 on the TIME, HER2 positive biopsies from the TRAP supplemented with the HER2 positive biopsies from the control cohort (*n* = 44) were compared to HER2 negative biopsies (*n* = 40; *n* = 39 eligible for analysis) from the control cohort. The study was performed in accordance to the guidelines for Good Clinical Practice and the Declaration of Helsinki. Written informed consent was provided by all participants or was previously obtained through the biobank for the control samples.

### Treatment

All patients (*N* = 84) received nCRT with paclitaxel (50 mg/m^2^), carboplatin (area under the curve=2), and radiotherapy (23×1.8 Gray over five weeks) according to the CROSS-regimen [4]. In addition, patients from the TRAP study (*n* = 40) received 4 mg kg^-1^ of trastuzumab on day 1, 2 mg kg^-1^ per week during weeks 2 to 6, and 6 mg kg^-1^ per week during weeks 7, 10 and 13, and 840 mg of pertuzumab every 3 weeks for a total of 5 administrations [12]. Both trastuzumab and pertuzumab were administered intravenously over 90 and 60 min, respectively, in the first cycle, followed by both 30 min in subsequent cycles.

### RNA isolation and gene expression analysis

Formalin-fixed paraffin embedded pre-treatment biopsies from the primary tumor were cut into slides of 4 µm. A minimum input of 8 slides and a maximum input of 18 slides per biopsy (mean 13 slides) were used for RNA isolation, depending on the availability of slides. RNA was isolated using the RNeasy FFPE kit (Qiagen, Hilden, Germany) as per the instructions and stored at −80 °C (Supplementary Figure 1). RNA concentration and integrity were determined using Nanodrop (ThermoFisher, Waltham, MA) and a 2100 BioAnalyzer (Agilent Technologies, Santa Clara, CA). The NanoString nCounter PanCancer IO360 panel (XT-CSO—HIO360–12) was used to profile the samples. The panel consisted of 770 genes, including 20 internal reference genes. In short, a total of 50 ng/5 µL RNA was added to the hybridization buffer and the Reporter and Capture probeset, and the samples were hybridized for 20 h in 65 °C. The hybridized samples were analysed using the NanoString nCounter SPRINT profiler with 12 samples per run. First, the assay performance of the data was assessed through quality control metrics using imaging quality control, binding density quality control, positive control linearity quality control and limit of detection. Secondly, raw data were normalized using the nCounter Advanced Analysis 2.0 module of NanoString (NanoString, Seattle, WA, USA) normalizing for technical variability using positive control normalization and assay input variability using codeset content normalization with the housekeeping genes. A selection of the most stable housekeeping genes for normalization was selected using the geNorm algorithm for mRNA [22]. Data with low expression below the set threshold (count value of 20 or observation frequency <0.5) were filtered (Supplementary Methods). Finally, the Advanced Analysis module of NanoString and the R2 platform for gene expression (http://r2.amc.nl) were used for data analyses. For pathway analyses, we used the Hallmark (2020) and KEGG signatures. Immune cell subset analyses were performed using the Cell Type Profiling from NanoString, analysing the normalized expression of genes associated to specific immune cell subtypes. The total tumor infiltrating lymphocyte (TIL) score is calculated using the average cell scores of B-cells, T-cells, macrophages, and cytotoxic cells.

### Statistical analysis

Progression-free (PFS) and overall survival (OS) were estimated using Kaplan-Meier analyses, calculated from treatment initiation to documented progression or death of any cause, respectively. The Student's *t*-test or MannWhitney U test were used for statistical analyses according to the data distribution, with a *p*-value <0.05 considered as statistically significant. A correction for multiple testing was applied using Bonferroni.

## Results

### Baseline characteristics

**Fig. 1:**
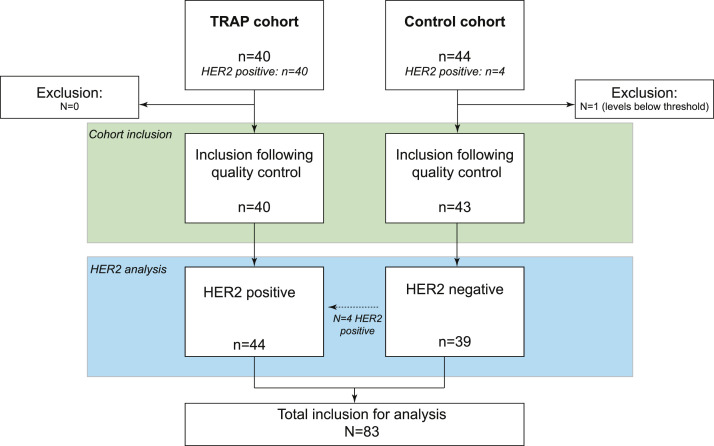
**Inclusion of biopsies**. Flow chart of baseline biopsies inclusion from the TRAP and control cohort.

In total, biopsies from 84 patients were included in this study; 83 biopsies were eligible for analyses following quality control (Fig. 1; TRAP cohort *n* = 40, control cohort *n* = 43). Baseline biopsies from all 40 patients in the TRAP cohort were included, of whom 83 % were male with a median age of 63 years (range, 45–78). 11 patients had HER2 2^+^ determined by immunohistochemistry with *ERBB2* gene amplification based on ISH and 29 patients had HER2 3^+^ positive tumors. Baseline biopsies from 43 patients of the control cohort were included, of whom 91 % were male with a median age of 65 years (range, 33–79); 4 patients had HER2 3^+^ positive tumors according to IHC, 39 patients had HER2 negative tumors. No significant differences in baseline clinical characteristics were identified between the TRAP and control cohort (Table 1) or HER2 positive biopsies and HER2 negative biopsies (Supplementary Table 1).

**Table 1:** Baseline characteristics of all included biopsies.

Characteristic	*N* = 40	*N* = 43	Statistics
	TRAP No (%)	Control No (%)	*P*-value
**Age** Median (years)	63	65	0.466
Range	45–78	33–79	
**Sex**			0.276
Male	33 (83 %)	39 (91 %)	
Female	7 (18 %)	4 (9 %)	
**HER2**			<0.001
HER2 positive	40 (100 %)	4 (9 %)	
HER2 2^+^ + SISH	11 (28 %)	0 (0 %)	
HER2 3^+^	29 (73 %)	4 (9 %)	
**Treatment**			<0.001
CROSS + *T*/P	40 (100 %)	0 (0 %)	
CROSS	0 (0 %)	43 (100 %)	
**Resection**			
Yes	38 (95 %)	40 (93 %)	0.709
R0	38 (100 %)	38 (95 %)	0.156
**Mandard score**			0.176
1	13 (33 %)	5 (12 %)	
2	10 (25 %)	11 (26 %)	
3	10 (25 %)	16 (37 %)	
4	3 (8 %)	7 (16 %)	
5	2 (5 %)	1 (2 %)	
NA	2 (5 %)	3 (7 %)	
**Age** Median (years)	63	65	0.466
Range	45–78	33–79	
**Sex**			0.276
Male	33 (83 %)	39 (91 %)	
Female	7 (18 %)	4 (9 %)	
**HER2**			<0.001
HER2 positive	40 (100 %)	4 (9 %)	
HER2 2^+^ + SISH	11 (28 %)	0 (0 %)	
HER2 3^+^	29 (73 %)	4 (9 %)	
**Treatment**			<0.001
CROSS + *T*/P	40 (100 %)	0 (0 %)	
CROSS	0 (0 %)	43 (100 %)	
**Resection**			
Yes	38 (95 %)	40 (93 %)	0.709
R0	38 (100 %)	38 (95 %)	0.156
**Mandard score**			0.176
1	13 (33 %)	5 (12 %)	
2	10 (25 %)	11 (26 %)	
3	10 (25 %)	16 (37 %)	
4	3 (8 %)	7 (16 %)	
5	2 (5 %)	1 (2 %)	
NA	2 (5 %)	3 (7 %)	

*43 patients were included in the control cohort following quality control selection (1 sample was excluded due to expression levels below the threshold).

### HER2 expression associates with epithelial tumor characteristics

**Fig. 2:**
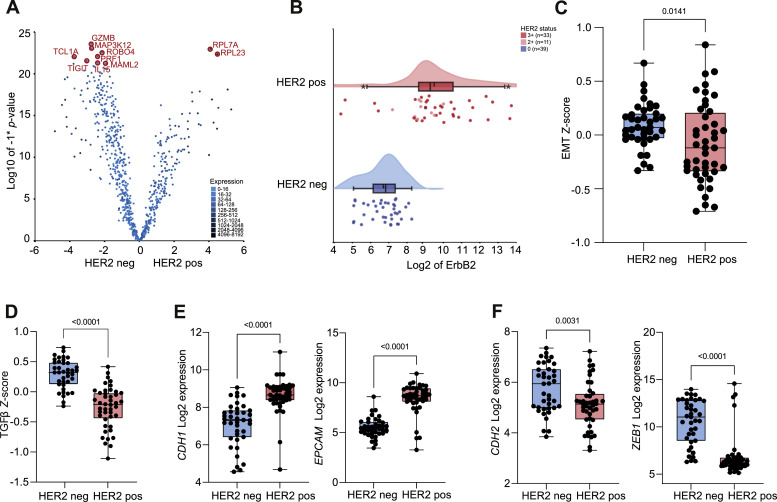
**HER2 associates with epithelial tumor characteristics.** A) Volcano plot of differentially expressed genes in patients with HER2 negative and HER2 positive biopsies; marked are the top 10 differentially expressed genes. B) Raincloud plot demonstrating correlation between *ERBB2* gene expression (Nanostring) and HER2 expression (positive vs. negative) and HER2 status according to immunohistochemistry. C) *Z*-score expression of epithelial-to-mesenchymal-transition (EMT) pathway between HER2 negative vs. HER2 positive biopsies, with lower expression in HER2 positive biopsies. D) Difference in expression of Z-score of TGFβ pathway between HER2 negative vs. HER2 positive biopsies, with lower expression in HER2 positive biopsies. E) Higher expression of epithelial-like targets *CDH1* and *EPCAM* in HER2 positive biopsies compared to HER2 negative biopsies. F) Lower expression of mesenchymal-related targets *CDH2* and *ZEB1* in HER2 positive biopsies compared to HER2 negative biopsies.

We first assessed the differential gene expression between HER2 positive (*n* = 44) and HER2 negative (*n* = 39) biopsies. The number of differentially expressed genes was well-balanced between the HER2 positive and HER2 negative cohort (Fig. 2A; top 20 differentially expressed genes are shown in Supplementary Table 2). *ERBB2* gene expression significantly correlated with HER2 expression as determined by IHC (Fig. 2B; *p* < 0.001), with higher gene expression in biopsies wit IHC 3+ compared to IHC 2+.

We found that HER2 expression associates with less epithelial-to-mesenchymal transition (EMT). First, a lower expression of the EMT geneset was observed in HER2 positive biopsies (Supplementary Figure 2). However, we noted that this was possibly confounded by lower expression of stromal genes in the HER2 positive cohort (Supplementary Figure 3). Therefore, we defined a geneset of tumor cell-intrinsic mesenchymal markers by subtracting genes of the ESTIMATE stroma geneset from the Hallmark EMT geneset (Supplementary Table 3) [23]. The geneset of tumor cell-intrinsic mesenchymal markers demonstrated lower expression in HER2 positive biopsies compared to the HER2 negative samples (Fig. 2C). Second, it has been suggested that EMT could be promoted through activation of the transforming growth factor β (TGFβ) pathway [24]. Similarly, we found a significantly lower expression of the TGFβ pathway geneset in HER2 positive tumors (*p* < 0.001; Fig. 2D). Third, decreased expression of individual tumor-intrinsic mesenchymal markers was confirmed in HER2 positive biopsies; mesenchymal markers such as *CDH2, ZEB1*, and *SNAI1* showed higher expression in the HER2 negative cohort (*p* < 0.001; Fig. 2E). In the HER2 positive samples, epithelial markers such as *EPCAM* and *CDH1* (*E-cadherin*) demonstrated higher expression (*p* < 0.001; Fig. 2F).

### HER2 positive tumors feature reduced immune cell infiltration

It has previously been shown that both TGFβ and mesenchymal tumor features contribute to immune evasion through upregulation of inhibitory signals that contribute to immune exhaustion, such as PD-L1, stimulation of immune suppressive factors, and inhibition of antigen presentation [25,26]. Thus, we assessed potential differences in the immune microenvironment between the HER2 positive (*n* = 44) and HER2 negative (*n* = 39) biopsies. Herein, we found that HER2 positive tumors feature reduced immune cell infiltration.

**Fig. 3:**
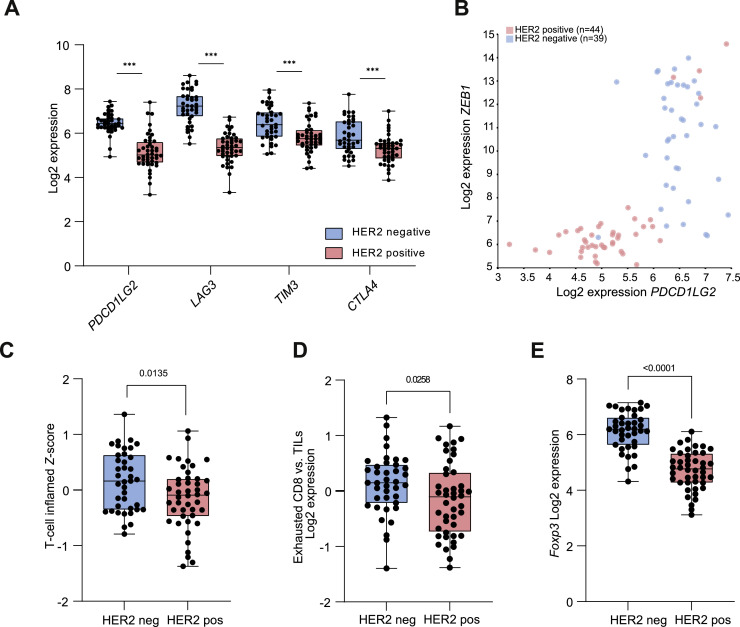
**HER2 expression features reduced tumor immune cell infiltration and immune exhaustion.** A) Higher expression of immune exhaustion markers *PDCD1LG2, LAG3, TIM3* and *CTLA4* in HER2 negative vs. HER2 positive biopsies. B) Higher T-cell inflamed score in HER2 negative biopsies vs. HER2 positive biopsies[29]. C) Gene vs gene correlation showing the positive correlation of the Log2 expression of *ZEB1* and *PDCD1LG2* (*r* = 0.676, *p* < 0.001) with HER2 positive samples in pink (*n* = 44) and HER2 negative samples in blue (*n* = 39). D) Higher expression of exhausted CD8 cell subsets in relation to total tumor infiltrating lymphocyte (TIL) count in HER2 negative biopsies vs. HER2 positive biopsies. E) Correlation between *FOXP3* expression and HER2 positivity vs. HER2 negativity, with inverse correlation between *FOXP3* expression and HER2 expression.

First, a distinctly different expression of previously annotated immune exhaustion markers was identified between HER2 positive and HER2 negative biopsies. A lower expression of *PDCD1LG2, LAG3, TIM3 and CTLA4* was observed in the HER2 positive biopsies (*p* < 0.001; Fig. 3A) [27]. Moreover, both *PDCD1LG2* and *LAG3* showed a statistically significant positive correlation with the mesenchymal markers *ZEB1* (*r* = 0.676; *r* = 0.574) and *SNAI1* (*r* = 0.663; *r* = 0.540), whereas a statistically significant negative correlation was observed with *EPCAM* (*r*=−0.831; *r*=−0.735) or *CDH1* (*r*=−0.590; *r*=−0.438), respectively. Thus, it could be hypothesized that the mesenchymal features of HER2 negative biopsies may induce immune suppression through upregulation of immune exhaustion, whilst the opposite is observed in the HER2 positive biopsies with increased epithelial features (Fig. 3B; Supplementary Figure 4).

To identify the potential effect of HER2 on immune cell infiltration, we investigated differences in immune cell infiltration between HER2 positive vs. HER2 negative biopsies. First, a significantly lower expression of the total TIL score was identified in the HER2 positive cohort (*p* < 0.01). Therefore, we analysed the ratio of immune cell subtypes relative to the total TIL score. HER2 positive biopsies had significantly lower infiltration of B-cells (*p* < 0.001), dendritic cells (*p* < 0.001), NK-cells (*p* < 0.001), and CD8 T-cells (*p* < 0.001) relative to the total TIL score compared to HER2 negative samples (Supplementary Figure 5). No significant difference was found in the infiltration of macrophages or neutrophils (*p* > 0.05) between HER2 positive and HER2 negative samples. Moreover, the expression of a validated T-cell inflamed score was significantly lower in the HER2 positive compared to the HER2 negative cohort (*p* = 0.0135; Fig. 3C) [28]. Thus, lower immune cell infiltration was observed in HER2 positive tumors. To confirm this, we analysed the expression of the nanostring geneset ‘Cytotoxic T-cell’, as well as the expression of individual molecular signals associated with immune activation. Indeed, the expression of the cytotoxic T-cell geneset was lower in HER2 positive tumors (Supplementary Figure 6), as well as a statistically significantly reduced expression of interleukine-10 (*IL-10; p* < 0.001), TGFβ2 (*p* < 0.001) and TGFβ3 (*p* < 0.001). Hence, this corroborates the finding that HER2 associates with reduced immune cell infiltration.

We hypothesized that since we found a decreased expression of immune exhaustion markers in HER2 positive biopsies, the abundance of exhausted CD8 T-cells according to cell type profiling would be reduced. And indeed, less exhausted CD8 T-cells were observed relatively to the total TIL score in the HER2 positive compared to the HER2 negative cohort (Fig. 3D).

Along these lines, the expression of *FOXP3*, one of the main markers of regulatory T-cells (Treg), was significantly lower in HER2 positive samples (*p* < 0.001; Fig. 3E) [26,29]. Unfortunately, markers of specific Treg cell subsets were below detection of threshold.

Overall, these data suggest that HER2 positive biopsies feature reduced immune cell infiltration and lower expression of inhibitory signals that associate with immune exhaustion, which could together indicate a less inflamed TIME.

### Immune exhaustion predicts resistance to anti-HER2 treatment

**Fig. 4:**
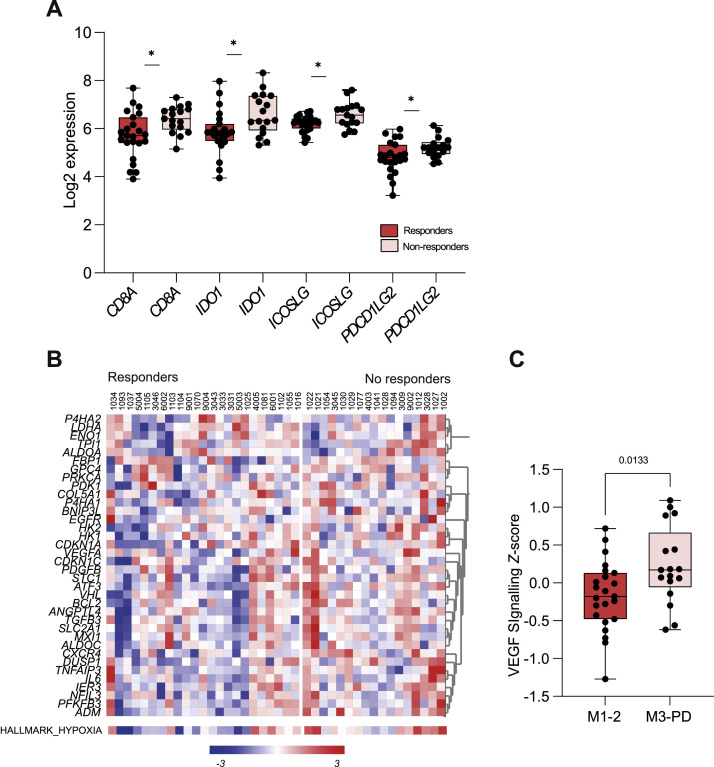
**Biomarkers associated with response to anti-HER2 treatment.** A) Lower expression of immune exhaustion markers (*CD8A, IDO1, ICOSLG, PDCD1LG2*) in responders (mandard 1–2, *n* = 23) vs. non-responders (mandard 3–5 and progressive disease before resection, *n* = 17). B) Heatmap of expression of the hypoxia pathway showing higher expression in non-responders (mandard 3–5 and progressive disease, *n* = 17) vs. responders (mandard 1–2, *n* = 23). C) Higher expression of *Z*-score of the VEGF-signalling pathway in non-responders (mandard 3–5 and progressive disease) vs. responders (mandard 1–2; *p* = 0.013).

Upregulation of immune exhaustion markers can induce T-cell exhaustion, interfering with the ability of T-cells to activate anti-tumor immunity [30]. Thus, we hypothesized that upregulation of inhibitory signals associated with immune exhaustion could confer resistance to anti-HER2 treatment. We used gene expression data from the baseline biopsies of the HER2 positive TRAP cohort (*n* = 40) treated with dual-agent HER2 targeted treatment to compare differences between patients with response (Mandard 1–2; *n* = 23) to patients with limited or no response (Mandard 3–5 and progression of disease prior to resection; *n* = 17) [31]. Indeed, the majority of upregulated genes in non-responders were associated with immune exhaustion (*ICOSLG, IDO1, CD8A, PDCD1LG2, CXCR4; p* < 0.05; Fig. 4A). Of note, no significant difference in immune cell infiltration could be identified between patients with response to HER2 targeting vs. no response (B-cells, dendritic cells, mast cells, neutrophils, NK-cells, CD8 T-cells; *p* > 0.05, Supplementary Figure 7), except for an overall increase of TILs in non-responders (*p* = 0.029). Thus, our data suggest that there is limited difference in baseline immune cell infiltration between responders and non-responders, and baseline immune infiltration does not hold value in prediction of response to treatment. However, as the expression of immune exhaustion markers at baseline associated with non-response, these could serve as therapeutic targets to HER2 targeted treatment.

In addition to immune-related expression we also assessed additional potential candidate therapeutic targets by assessing differential expression between responders vs. no responders. The top differentially expressed gene was vascular endothelial growth factor B (*VEGFB*), with higher expression in non-responders (*p* = 0.004; Supplementary Table 4). Similarly, pathway analyses demonstrated higher expression of signatures for hypoxia (Fig. 4B), angiogenesis, and VEGF signalling (Fig. 4C) in non-responders compared to responders at baseline. Several hypoxia-related genes, such as *ATF3, ANGPTL4*, and *CXCR4*, were indeed significantly upregulated in non-responders (*p* < 0.05). Thus, we demonstrated higher expression of immune exhaustion markers, as well as increased expression of hypoxia and VEGF related markers at baseline as potential therapeutic targets in non-responders. VEGF could potentially exert immunosuppressive features, as previously shown [32]. This suggests that the addition of anti-VEGF agents to anti-HER2 targets could hold potential value to reduce treatment resistance.

## Discussion

In this study we demonstrated that HER2 drives an epithelial phenotype and distinctive TIME profile with lower immune cell infiltration and less expression of inhibitory signals associated with immune exhaustion compared to HER2 negative tumors. Moreover, lower expression of immune exhaustion markers and reduced VEGF expression correlated with increased response to anti-HER2 agents.

HER2 positivity in both HER2 positive EAC and breast cancer has been associated with epithelial tumor characteristics [33,34]. Preclinical studies have shown that HER2 inhibition leads to a decrease in epithelial markers [33,34]. This loss of epithelial phenotype enhances cancer cell migratory capacity through EMT, resulting in a poor disease outcome [35]. [36,37] Interestingly, HER2 targeting can reverse this process: when the receptor blockade is lifted, tumor cells can revert to their epithelial state [33]. This reversibility suggests that temporary discontinuation of anti-HER2 agents, known as ‘drug holidays’, could overcome treatment resistance caused by drug-induced EMT. However, the optimal balance between inhibiting HER2-induced oncogenesis and reverting induced mesenchymal features through HER2 blockade alleviation remains unknown.

Our group previously demonstrated an increased secretion of TGFβ receptor ligands upon HER2 inhibition, which also resulted in the loss of epithelial features in HER2 positive EAC xenografts [33]. Similarly, we found a reduced expression of TGFβ in HER2 positive tumors, which were associated with less EMT. This suggests that TGFβ expression could drive EMT [24]. Both in vitro and in vivo studies demonstrated increased anti-tumor response from the addition of TGFβ inhibition to anti-HER2 targeted compounds [33,38,39]. Further studies should evaluate if response to anti-HER2 treatment could be enhanced through the addition of TGFβ targeting agents in HER2 positive EAC.

Recent findings of a large comparative analysis in HER2 positive gastroesophageal carcinoma demonstrated lower immune cell infiltration at baseline in high HER2 overexpressing tumors [17]. Several other smaller studies have reported contradictory results in HER2 positive gastroesophageal tumors, showing an increase in immune cell infiltration and TIL expression compared to HER2 negative tumors [40,41]. Our findings indicate a reduced immune cell infiltration in HER2 positive EAC, but also a reduction in immune exhaustion markers. As ADCC is postulated as one of the main mechanisms of HER2 targeting, one could expect reduced sensitivity to anti-HER2 treatment of tumors associated with lower immune cell infiltration and less immune exhaustion [13]. It could be hypothesized that the infiltrated immune cells present in the TIME of the HER2 positive tumors are activated upon anti-HER2 treatment to trigger anti-tumor immunity, especially since we found lower expression of inhibitory signals associated to immune exhaustion. Likewise, a previous preclinical study demonstrated an increase in TIL following two weeks of anti-HER2 treatment [42]. This would also be in line with the positive outcomes of previous clinical studies combining immune checkpoint inhibitors with anti-HER2 agents in advanced positive GEC and breast cancer [[43], [44], [45]]. The first interim results from the KEYNOTE-811 demonstrated a significant increase in response rate with pembrolizumab, trastuzumab and chemotherapy for advanced gastroesophageal adenocarcinoma compared to trastuzumab and chemotherapy alone [43]. Nevertheless, no large differences in duration of response were observed between both cohorts, with a median response duration of 10.6 months with pembrolizumab and trastuzumab compared to 9.5 months with trastuzumab only. Taking into account the potential limited duration of response and our findings of low immune cell infiltration in HER2 positive EAC, it remains unclear if the response triggered by anti-tumor immunity in HER2 positive gastroesophageal adenocarcinoma will result in clinically relevant survival benefit. The survival results of the ongoing KEYNOTE-811 are eagerly awaited.

We found an increased signature expression of both VEGF and hypoxia in non-responders within the TRAP cohort, as well as increased *VEGFB* expression. It has previously been reported that VEGF could also exert immunosuppressive features [32]. Since we also identified immune exhaustion as potential therapeutic target in non-responders at baseline, our data indeed suggests that increased VEGF expression could induce immunosuppression, and thus less response to anti-HER2 agents. Therefore, these patients could benefit from combining anti-VEGF agents to HER2 targeted treatment. In *vivo* studies demonstrated greater tumor inhibition by combining anti-VEGF and anti-HER2 antibodies in a gastric xenograft model compared to both compounds individually [46]. Encouraging results have been demonstrated in the clinical setting in HER2 positive esophagogastric cancer with the combination of VEGF and HER2 targeting [47]. Further research is warranted to investigate potential synergism of these compounds in HER2 positive EAC.

Several limitations merit closer attention. First, we included a relatively small cohort with solely baseline biopsies. Therefore, results remain exploratory, and treatment specific effects cannot fully be evaluated. Secondly, by performing Nanostring analyses only a subset of targets focussing on immune-oncology was assessed. Third, by the addition of archival tissues the two cohorts a selection bias could have been introduced, although normalization has been performed. Fourth, we investigated gene expression patterns in tumor biopsies. Future research including functional experiments to confirm the association between HER2 and a specific immune microenvironment requires an appropriate immunocompetent in vivo model to corroborate our findings. The insights from our study could guide the development of appropriate in vivo models, capturing the true complexity of the tumor microenvironment interactions in HER2 positive esophageal adenocarcinoma. Moreover, future studies should increase the sample size, allowing for deeper comprehension of the molecular mechanisms between the signal transduction pathways in HER2 positive vs. HER2 negative tumors.

In conclusion, HER2 expression associated with epithelial tumor characteristics. Moreover, HER2 positive EAC feature reduced immune cell infiltration, but also less immune exhaustion. Taking into account a less inflamed TIME in HER2 positive tumors, it remains to be elucidated if co-administration of immune checkpoint inhibitors and anti-HER2 targeting result in clinically relevant benefit. As limited response was associated with increased expression of VEGF, clinical studies are warranted to investigate potential synergism of combining VEGF and HER2 targeting.

## Data availability

The sequence data generated in this study is made publicly available in the Gene Expression Omnibus database (GEO, GSE274220), and is available within the article and its supplementary data files. Additional clinical data will be made available upon request for academic researchers with a scientific research question.

## Funding

Funding for this investigator-initiated study was derived from the Amsterdam UMC, location AMC, without financial support from external sponsors.

## CRediT authorship contribution statement

**Charlotte I. Stroes:** Writing – review & editing, Writing – original draft, Visualization, Methodology, Formal analysis, Data curation, Conceptualization. **Sybren L. Meijer:** Writing – review & editing, Data curation, Conceptualization. **Geert-Jan Creemers:** Conceptualization, Writing – review & editing, Data curation. **Gerrit K.J. Hooijer:** Writing – review & editing, Data curation, Conceptualization. **Nadia Haj Mohammad:** Writing – review & editing, Data curation, Conceptualization. **Maartje Los:** Writing – review & editing, Data curation, Conceptualization. **Marije Slingerland:** Writing – review & editing, Data curation, Conceptualization. **Geke A.P. Hospers:** Writing – review & editing, Data curation, Conceptualization. **Annemieke Cats:** Writing – review & editing, Data curation, Conceptualization. **Laurens V. Beerepoot:** Writing – review & editing, Data curation, Conceptualization. **Maarten F. Bijlsma:** Writing – review & editing, Writing – original draft, Supervision, Methodology, Data curation, Conceptualization. **Hanneke W.M. van Laarhoven:** Writing – review & editing, Writing – original draft, Supervision, Resources, Project administration, Methodology, Formal analysis, Data curation, Conceptualization.

## Declaration of competing interest

All authors completed the disclosure of conflict of interest. The following authors declared a potential conflict of interest. M. Slingerland reports an advisory role for Lilly, AstraZeneca and BMS. N. Haj Mohammad reports an advisory role for BMS, Merck, Servier, Elli Lilly, and AstraZeneca, and has received honoraria for lectures from Servier and BMS with fees paid to the institution. G.K. Hospers reports a consultancy/advisory role for Amgen, Bristol-Myers Squibb, Roche, MSD, Pfizer, Novartis, Sanofi, Pierre Fabre, and has received research grants from Bristol-Myers Squibb, Seerave. L. Beerepoot reports an advisory role for Servier, Ipsen, Medtalks and Travel Congress Management, and reports a leadership role for Ciebom NVMO and cieOOM NVMO. M.F. Bijlsma has received research funding from Celgene, Frame Therapeutics and Lead Pharma, and has acted as a consultant to Servier and Olympus. H.W.M. van Laarhoven reports an advisory role for AMphera, Anocca, Astellas, AstraZeneca, Beigene, Boehringer, Daiichy-Sankyo, Dragonfly, MSD, Myeloid, Servier, has received research funding, medication supply and/or research support from Auristone, Incyte, Merck, ORCA and Servier, and has had a speaker role at Astellas, Beigene, Benecke, BMS, Daiichy-Sankyo, JAAP, Medtalks, Novartis, Springer, Travel Congress Management BV. All fees were paid to the institution.

## Appendix Supplementary materials

Image, application 1
