# Supplementary material for: Tumor immune microenvironmental characteristics in Human Epidermal Growth Factor-2 (HER2) positive esophageal adenocarcinoma: A comparative analysis and biomarker study
Source: Transl Oncol. 2024 Aug 15;49:102079. doi: 10.1016/j.tranon.2024.102079 (PMC11375138; doi:10.1016/j.tranon.2024.102079)
Supplement: Supplementary file 1 [file mmc1.pdf]

SUPPLEMENT

Normalization

The gene expression data were uploaded in the nSolver program from nanostring. First, the assay performance was evaluated using quality control metrics; a detailed description can be found in the Gene Expression Data Analysis Guidelines (Nanostring, MAN-C0011-04).

In short, quality control metrics were assessed for each sample:

- Imaging quality: each individual lane scanned on an nCounter system is divided into a few hundred imaging sections, called fields of view (FOV). The number of FOVs successfully imaged is reported as FOV Counted. Imaging performance could be insufficient if a discrepancy occurs between the FOV counted and the number of FOV for which imaging was attempted. Thus, this ratio should be at least higher than 75% in order to reach sufficient imaging quality.
- Binding density quality: the imaging unit should count codes that are unambiguously distinguishable. If codes overlap it could cause image saturation, which could eventually lead to data loss. Thus, the binding density quality is evaluated by calculating the number of optical features per square micron for each lane (range 0.1-1.8). If the binding density of a sample is within this range, the binding density is sufficient. Factors that could influence the binding density are assay input quantity and the expression level of genes.
- Positive control linearity quality: in each nanostring assay six synthetic DNA control targets are included in order to measure the efficiency of the hybridization reaction. Since the concentrations of the control targets range linearly, the R2 value of the known Log2 concentration of the input of the positive controls divided by the measured Log2 concentration of the positive controls determines the positive control linearity. If the R2 value is above 0.95, samples demonstrate sufficient positive control linearity quality.
- Limit of detection quality: the limit of detection was evaluated by assessing the counts of one of the positive control targets (POS_E), which should be higher than two times the standard deviation above the mean of the negative controls included in the assay. Factors that could influence the limit of detection are too high background noise or very low counts due to suboptimal hybridization.

If samples passed the quality control, raw data were included in analyses. Data were normalized using the nCounter Advanced Analysis 2.0. Detailed information can be found in the nanostring nCounter Advanced Analysis 2.0 User Manual (MAN-10030-30). Raw data were normalized for technical variability using positive control normalization and assay input variability using codeset

content normalization with the housekeeping genes. A selection of the most stable housekeeping genes for normalization was selected using the geNorm algorithm for mRNA.1 Covariates used for normalization in analysis were cohorts, Mandard score, cartridge ID, scanned data, FOV Counted, binding density, and imaging quality.

Supplementary Table 1 | Baseline characteristics of HER2 positive samples (n=44) vs HER2 negative samples (n=39)

Characteristic	N=44<br>HER2 positive No. (%)	N=39<br>HER2 negative No. (%)	Statistics<br>*P*-value
**Age**			0.286
Median (years)	63.0	66.0	
Range	45-78	33-79	
**Sex**			0.381
Male	37 (84%)	35 (90%)	
Female	7 (16%)	4 (10%)	
**HER2**			<0.001
HER2 positive	44 (100%)	0 (0%)	
HER2 2+ + SISH	11 (25%)	0 (0%)	
HER2 3+	33 (75%)	0 (0%)	
**Treatment**			<0.001
CROSS + T/P	40 (91%)	0 (0%)	
CROSS	4 (9%)	39 (100%)	
**Resection**			
Yes	42 (95%)	36 (92%)	0.553
R0	42 (100%)	34 (94%)	0.125
**Mandard score**			0.100
1	13 (30%)	5 (13%)	
2	13 (30%)	8 (21%)	
3	11 (25%)	15 (38%)	
4	3 (7%)	7 (18%)	
5	2 (5%)	1 (3%)	
NA	2 (5%)	3 (8%)	

Supplementary Table 2 | Top 20 differentially expressed genes between HER2-positive and HER2-negative baseline biopsies

HER2 positive > HER2 negative		HER2 negative > HER2 positive	
**Genes**	***P*-value**	**Genes**	***P*-value**
RPL7A	<0.001	*GZMB*	<0.001
RPL23	<0.001	*MAP3K12*	<0.001
LYZ	<0.001	*ROBO4*	<0.001
HLA-E	<0.001	*PRF1*	<0.001
NFKBIA	<0.001	*TCL1A*	<0.001
HLA-B	<0.001	*TIGIT*	<0.001
ENO1	<0.001	*IL15*	<0.001
PSMB5	<0.001	*MAML2*	<0.001
ALDOA	<0.001	*SOX10*	<0.001
B2M	<0.001	*MAGEC1*	<0.001
EPCAM	<0.001	*RORC*	<0.001
IFI27	<0.001	*STAT4*	<0.001
PKM	<0.001	*MICA*	<0.001
MKI67	<0.001	*PRKX*	<0.001
CTSS	<0.001	*CDC25C*	<0.001
CD74	<0.001	*TNFSF9*	<0.001
HLA-C	<0.001	*ITGB3*	<0.001
A2M	<0.001	*PLA1A*	<0.001
LAMC2	<0.001	*SLC11A1*	<0.001
LDHA	<0.001	*TWIST2*	<0.001

Supplementary Table 3 | Tumor-intrinsic epithelial to mesenchymal transition geneset

Genes			
TPM1	*COL5A1*	*PVR*	*LAMA1*
NOTCH2	*IL32*	*INHBA*	*TNFRSF11B*
TGFB1	*CD44*	*FSTL3*	*SFRP1*
ITGA2	*LAMC2*	*NID2*	*VEGFC*
IL6	*ITGAV*	*DKK1*	*TNFAIP3*
MMP1	*AREG*	*DAB2*	*ITGB3*
CXCL5	*NT5E*	*FZD5*	*WNT5A*
CXCL6	*SERPINH1*	*COL11A1*	*SPP1*
THBS1	*PLOD2*	*CDH2*	*VEGFA*
CDH11	*VCAN*	*GAS1*	
CXCL1	*THY1*	*LOXL1*	

Supplementary Table 4 | Top 20 differentially expressed genes between patients with limited response to treatment M3-PD (Mandard 3, 4, 5 and progression of disease) vs. M1-2 (Mandard 1 + 2)

**M3-PD > M1-2**	
**Genes**	**P-value**
VEGFB	0.004
CXCR4	0.007
RELA	0.008
APC	0.009
MICB	0.009
CD3E	0.010
HDAC4	0.010
ICOSLG	0.010
IDO1	0.011
SGK1	0.011
IFIT2	0.012
CTSW	0.014
CD8A	0.014
DUSP2	0.015
COMP	0.015
CCL5	0.015
WNT7B	0.015
HDAC3	0.016
MS4A4A	0.017

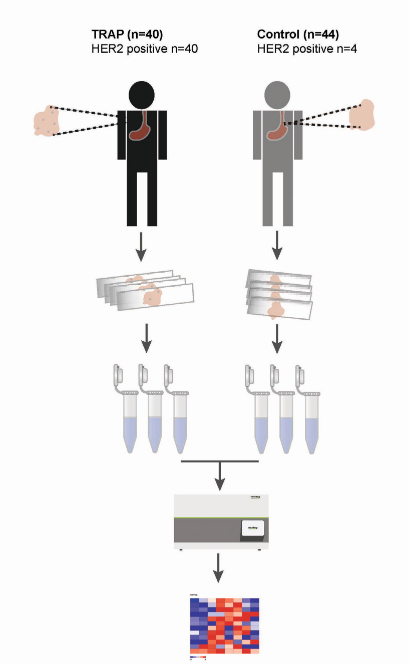

Supplementary Figure 1 | From RNA preparation to gene expression data via nanostring analyses.

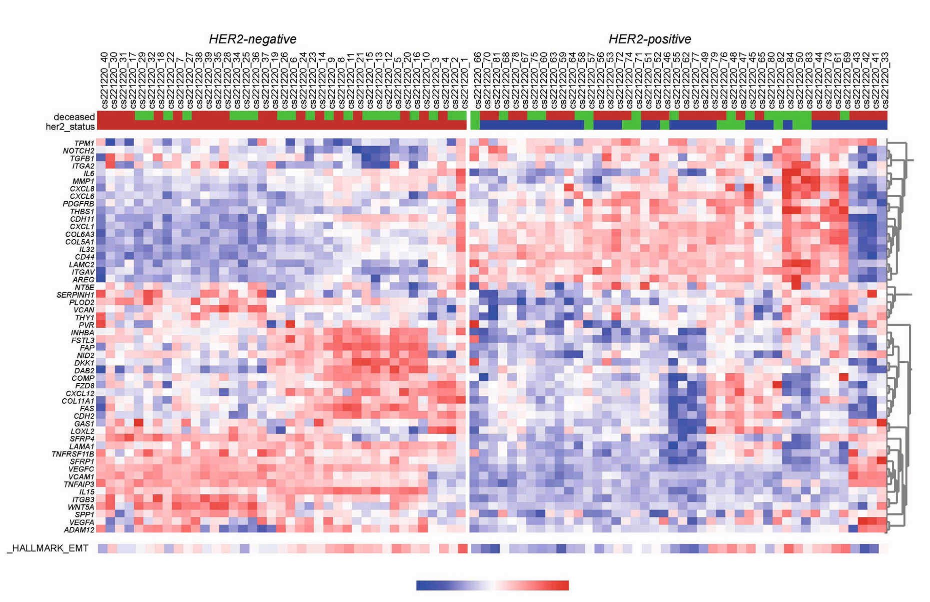

Supplementary Figure 2 | Heatmap of Epithelial to Mesenchymal Transition pathway (EMT; Hallmark 2020) showing a significantly higher expression in the cohort with HER2-positive tumors (n=44) vs. HER2-negative tumors (n=39; *p*=0.001)

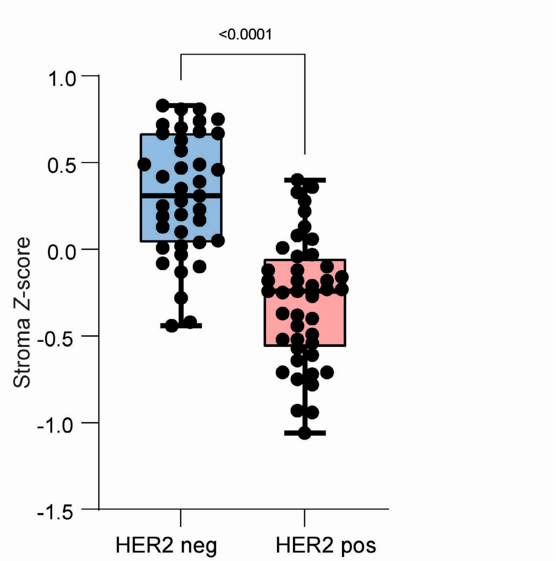

Supplementary Figure 3 | Expression of stroma geneset from ESTIMATE in patients with HER2 negative baseline biopsies (n=39) vs. HER2 positive baseline biopsies (n=44).

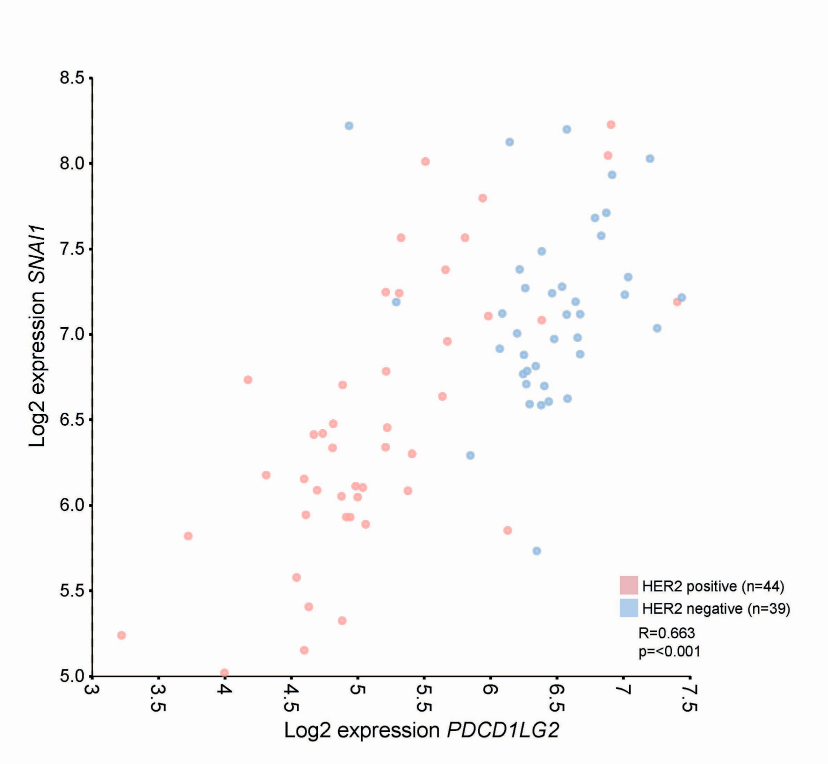

Supplementary Figure 4 | XY-plot showing Log2 expression of *SNAI1* and *PDCD1LG2* (R=0.662, *p*<0.001)

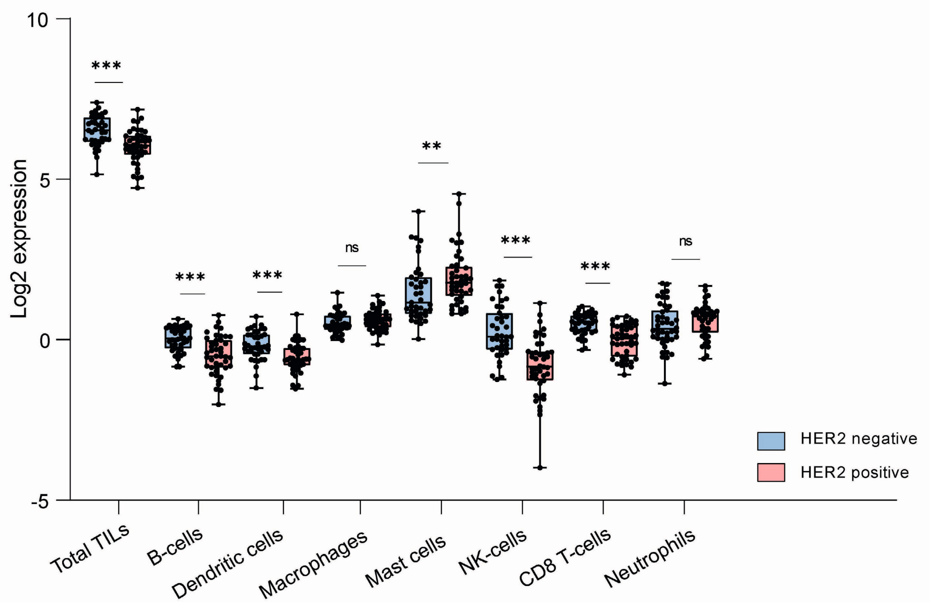

Supplementary Figure 5 | Boxplots of expression of immune cell subsets in patients with HER2 negative (n=39) vs. HER2 positive tumors (n=44). All immune cell subsets are relative to the total tumor infiltrating lymphocytes (TILs) score.

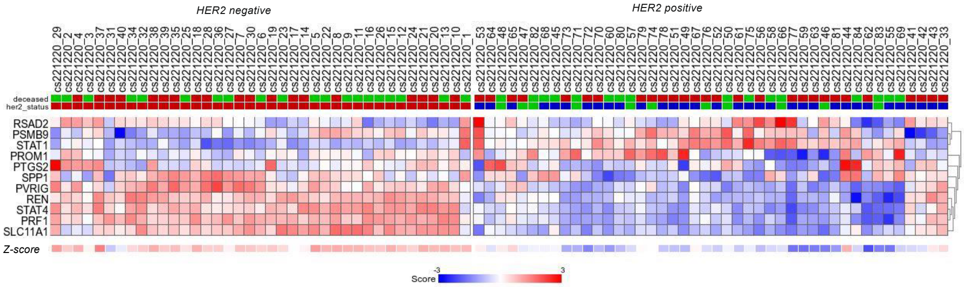

Supplementary Figure 6 | Expression of Nanostring Geneset 'Cytotoxic T-cells' in HER2 negatieve patients (red; left) vs. HER2 positive patients (blue; right), showing increased gene expression in patients with HER2 negative tumors.

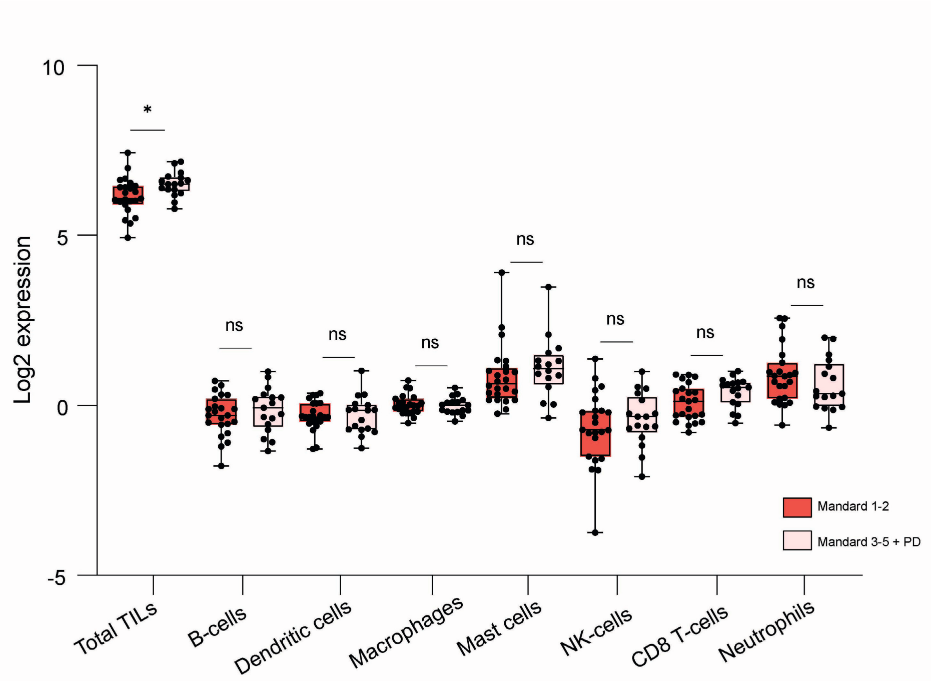

Supplementary Figure 7 | Boxplots of expression of immune cell subsets in patients with response (Mandard 1+2; n=23) vs. limited response (Manard 3-4-5 + progression of disease, n=17). All immune cell subsets are relative to the total tumor infiltrating lymphocytes (TIL) score.

REFERENCES

1 Vandesompele J, De Preter K, Pattyn F, et al: Accurate normalization of real-time quantitative RT- PCR data by geometric averaging of multiple internal control genes. Genome Biology 3: research0034.1, 2002

2 Yoshihara K, Shahmoradgoli M, Martínez E, et al: Inferring tumour purity and stromal and immune cell admixture from expression data. Nat Commun 4:2612, 2013
